# Controlling variation in the comet assay

**DOI:** 10.3389/fgene.2014.00359

**Published:** 2014-10-20

**Authors:** Andrew R. Collins, Naouale El Yamani, Yolanda Lorenzo, Sergey Shaposhnikov, Gunnar Brunborg, Amaya Azqueta

**Affiliations:** ^1^Department of Nutrition, Faculty of Medicine, University of OsloOslo, Norway; ^2^Department of Chemicals and Radiation, Norwegian Institute of Public HealthOslo, Norway; ^3^Department of Pharmacology and Toxicology, Faculty of Pharmacy, University of NavarraPamplona, Spain

**Keywords:** comet assay, variability, reference standards, biomonitoring, inter-laboratory comparisons

## Abstract

Variability of the comet assay is a serious issue, whether it occurs from experiment to experiment in the same laboratory, or between different laboratories analysing identical samples. Do we have to live with high variability, just because the comet assay is a biological assay rather than analytical chemistry? Numerous attempts have been made to limit variability by standardizing the assay protocol, and the critical steps in the assay have been identified; agarose concentration, duration of alkaline incubation, and electrophoresis conditions (time, temperature, and voltage gradient) are particularly important. Even when these are controlled, variation seems to be inevitable. It is helpful to include in experiments reference standards, i.e., cells with a known amount of specific damage to the DNA. They can be aliquots frozen from a single large batch of cells, either untreated (negative controls) or treated with, for example, H_2_O_2_ or X-rays to induce strand breaks (positive control for the basic assay), or photosensitiser plus light to oxidize guanine (positive control for Fpg- or OGG1-sensitive sites). Reference standards are especially valuable when performing a series of experiments over a long period—for example, analysing samples of white blood cells from a large human biomonitoring trial—to check that the assay is performing consistently, and to identify anomalous results necessitating a repeat experiment. The reference values of tail intensity can also be used to iron out small variations occurring from day to day. We present examples of the use of reference standards in human trials, both within one laboratory and between different laboratories, and describe procedures that can be used to control variation.

## Introduction

The comet assay is conventionally seen by many as a soft, biological assay, at best semi-quantitative. Variation does occur, between laboratories, and over time in the same laboratory, and so some form of standardization is advisable. It is in principle possible to improve the comparability of comet data by expressing results not just as % tail DNA, but as a frequency of DNA breaks, by calibrating the assay using cells that have been treated with different doses of X- or γ-radiation; it has been known since the days of alkaline sucrose gradient sedimentation that ionizing radiation induces damage in cellular DNA at the rate of 0.31 breaks per Gy per 10^9^ Dalton (Ahnstrom and Erixon, 1981). (Ionizing radiation is a very robust damaging agent, compared with chemicals, which can be greatly affected by the physico-chemical conditions of exposure, and particularly by the biological environment of enzymes and other molecules that can react with the chemical, decreasing or increasing its effectiveness—not to mention possible membrane barriers that can limit uptake.) This conversion to a “real” break frequency is not without its problems: most researchers do not have access to a radiation source, and so calibration tends to be second-hand, and historical. Even if a source is available, so that irradiated reference cells can be included in the same experiment, these cells and the sample cells are never assayed under the exact same conditions; they cannot be together in the same gel (as a true internal standard would be) unless some way is found to distinguish the two cell types after electrophoresis. (There are attempts to overcome this difficulty, as discussed later in this review.)

The comet assay is widely used in combination with formamidopyrimidine DNA glycosylase (Fpg) or 8-oxoGua DNA glycosylase (OGG1) to measure 8-oxoGua in DNA—an excellent marker of oxidative stress. In the mid-1990s, it became clear that estimates of the background level of 8-oxoGua in peripheral blood mononuclear (PBMN) cells from healthy subjects varied by orders of magnitude, depending on the assay employed. The comet assay + Fpg gave results on the low side, compared with chromatographic methods (HPLC, LC-MS.MS, GC-MS). In the ESCODD project (ESCODD, 2002, 2003; ESCODD et al., 2005) we set out to compare the different methods and decide which was most accurate. It turned out that the chromatographic methods were subject to oxidation of DNA during sample preparation, leading to a serious over-estimation of DNA base oxidation. The comet assay was free of this artifact. However, while the comet assay + Fpg was apparently more accurate than chromatography, it suffered—and still suffers—from lack of precision. ESCODD partners were sent identical cell samples to analyse, but the results varied greatly from laboratory to laboratory. By analogy with a game of darts, the comet assay results were clustered around the bull's eye, but did not score direct hits.

Here we will discuss the different levels of variation—experimental, inter-laboratory, intra-individual, inter-individual (or between different samples—e.g., different concentration of a chemical compound—in the case of experiments with cell culture), and even inter-national—that can be encountered with the comet assay. It is important to recognize—and limit as much as possible—the variation that arises from differences in experimental conditions, in order to maximize the variations that are of real interest, e.g., the variations between samples, subjects in a population study, or population groups in different countries. We will describe the measures that should be taken to ensure experimental consistency, encourage the use of reference standards, and suggest ways of accommodating experimental variation by normalization procedures.

## Identifying sources of variation

Trials have been carried out, in particular by the consortium known as ECVAG, specifically to examine variability in the comet assay, and to apply statistical analyses to quantitate the different sources of variation.

In the first ECVAG trial (Forchhammer et al., 2010), 12 laboratories received pre-made slides to score, a set of cryopreserved γ-irradiated cells to construct a standard curve, and a set of coded samples. The inter-laboratory coefficient of variation (CV) for the latter, 47%, was reduced to 28% when data were adjusted using the laboratory-specific standard curve. The second trial (Johansson et al., 2010) involved 10 laboratories and examined variation in the measurement of Fpg-sensitive sites; coded samples treated with Ro 19-8022 plus light were sent with a set of γ-irradiated reference samples. The inter-laboratory variation in assessment of Fpg-sensitive sites was mainly due to differences in protocols, and was decreased when standard curves (created from the reference samples) were used to adjust the results. The aim of the third ECVAG trial (Forchhammer et al., 2012) was to test the effect of introducing a standard protocol; several laboratories found it difficult to adopt the standard methods. Three coded human PBMN cell samples were analyzed: variation was very high, for strand breaks, and for Fpg-sensitive sites, and (in the case of Fpg-sensitive sites) was only slightly less with the standard protocol than with the laboratories' own protocols. The fourth trial (Ersson et al., 2013) set out to identify different sources of variation in analysis of real PBMN cell samples, and concluded that “inter-laboratory variation accounted for the largest fraction of the overall variation and the unexplained (residual) variation was much larger than the intra-laboratory variation…”

## Limiting variation

Differences in protocol seem to be largely responsible for variation between laboratories. Some likely sources of variation are obvious, but still deserve to be formally explored, and this was done independently by two groups a few years ago, with very similar conclusions. Ersson and Möller (2011) and Azqueta et al. (2011a) investigated the effect on comet formation (% tail DNA) of agarose concentration, duration of alkaline unwinding, electrophoresis period and voltage gradient. The % tail DNA of comets from cells treated with γ-rays (Ersson and Möller, 2011) or with H_2_O_2_ (Azqueta et al., 2011a) was greatest in 0.4% agarose (which is fragile, and not recommended), and steadily decreased with increasing concentration, up to >1%. The period of alkaline incubation before electrophoresis was varied, in both laboratories, up to 60 min, and steady increases in % tail DNA with time (at least up to 40 min) were seen in comets from cells treated with H_2_O_2_ (both laboratories) and also γ-irradiated cells and cells treated with photosensitiser plus light (to induce 8-oxoGua) and incubated after lysis with Fpg (Ersson and Möller, 2011). A similar dependence on time of alkaline unwinding was previously shown by Vijayalaxmi et al. (1992) and Speit et al. (1999). When the alkaline unwinding period was extended to 18 h, all DNA (from cells treated with N-methyl-N-nitro-N-nitrosoguanidine) was present in the tail (Yendle et al., 1997). The increase in tail intensity is likely to be due to conversion of alkali-labile sites (such as result from loss of bases from the DNA) to strand breaks.

It is generally assumed that the initial lysis in high salt and detergent is not critical, and lysis periods of 1 h, or overnight, or even days, or weeks, are common. A recent study (Enciso et al., in press) found that, for cells treated with methylmethanesulphonate or H_2_O_2_, similar values of % tail DNA were seen with 1 h of lysis or with no lysis at all (i.e., immediately placing slides into alkaline solution). Longer lysis periods, up to 1 week, led to an increase in % tail DNA, in the case of treated cells. However, if enzyme digestion is included in the comet assay procedure, for instance to detect 8-oxoGua, lysis is essential to make the DNA accessible to the enzyme; in this case, 5 min or 1 h of lysis gave similar results (Enciso et al., in press). Sensitivity (i.e., relative increase in % tail DNA in treated compared with untreated cells) was enhanced up to 24 h.

**Figure 1:**
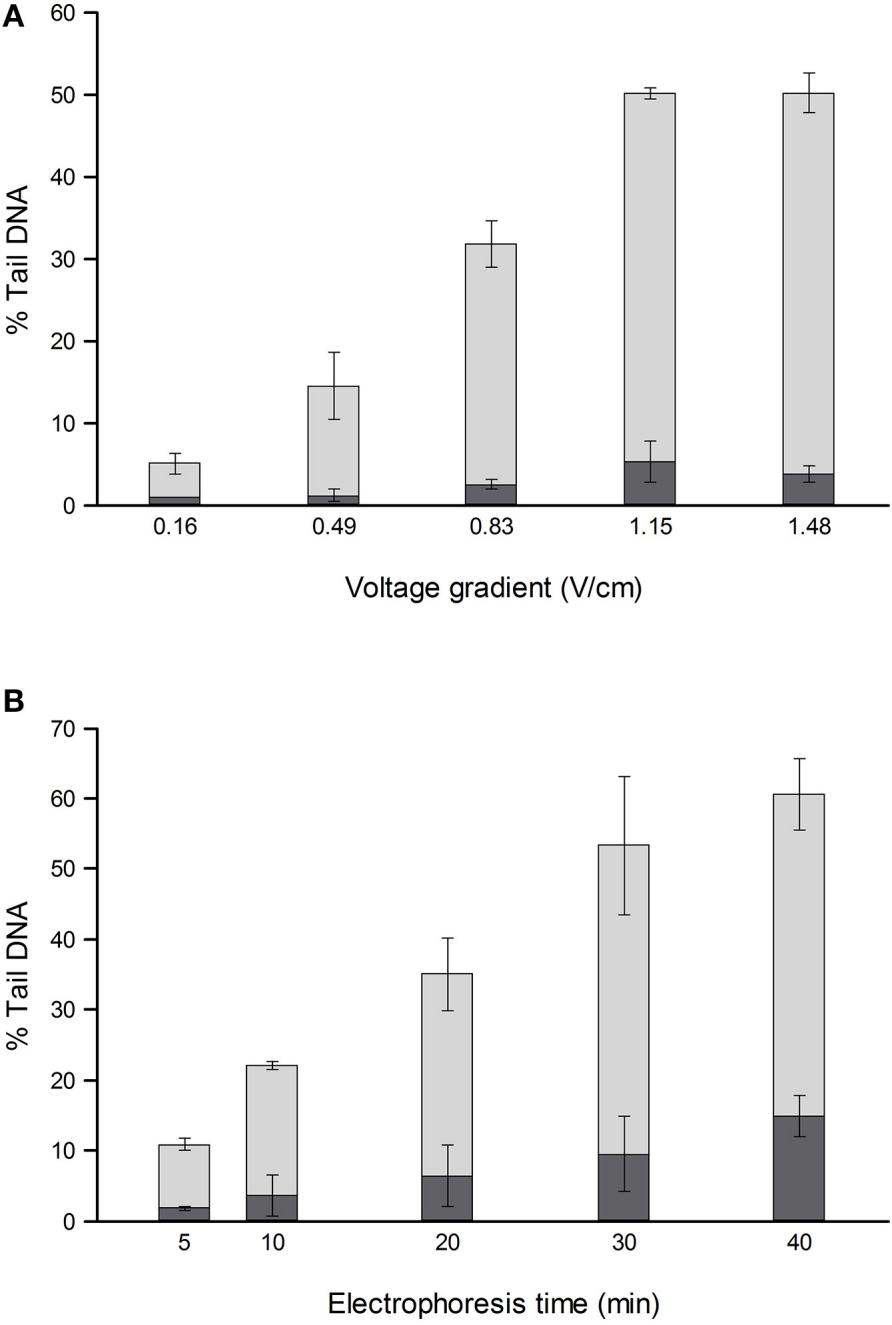
**Effect of different voltage gradients at constant current (A) and different electrophoresis times (B) on the % tail DNA in TK-6 cells, untreated (dark shading), and treated with 70 μM H_2_O_2_ (light shading)**. The mean and range of values from two experiments are shown. Redrawn from Azqueta et al. (2011a) with permission from Elsevier.

For comets from irradiated or H_2_O_2_-treated cells, the % tail DNA is strongly influenced by both electrophoresis time (varied up to 40 min) and voltage gradient (between <0.2 and 1.6 V/cm) (Ersson and Möller, 2011; Azqueta et al., 2011a) (Figure 1). To a certain extent, a low voltage gradient for a long time will give similar results to a higher voltage gradient for a shorter time. The voltage gradient should be measured over the platform on which the slides are placed rather than between the electrodes, since that is where the electric potential pulls out damaged DNA from the nucleoids. Between the electrode and platform edge, in standard tanks, there is a relatively deep trough of electrophoresis solution, with low resistance, so that the voltage drop is much lower than over the platform where there is a shallow layer of solution over the slides; hence, measuring the total applied voltage and dividing it by the distance between the electrodes gives an erroneous V/cm value. Increasing the depth of solution over the platform increases the current (because the resistance is decreased), and this causes a slight decrease in the % tail DNA—an effect explained by the reduced voltage drop over the platform (Azqueta et al., 2011a). Current itself does not influence DNA migration. These comments relate to tanks with electrodes in troughs and a central platform. There are few restrictions in tank design as long as the voltage gradient is constant where the samples are placed for electrophoresis. We recommend careful measurement of the voltage gradient at the relevant position and depth, particularly when non-standard electrophoresis tanks are being used.

Ersson and Möller (2011) looked also at the enzyme incubation step, measuring 8-oxoGua induced by photosensitiser Ro 19-8022 plus light. At a specific enzyme concentration, a maximum yield of DNA breaks (% tail DNA) was seen after 30 min digestion, with no increase at 45 min. This simply highlights the necessity to optimize incubation conditions for each batch of enzyme, whether obtained commercially or prepared in-house from an overproducing bacterial strain. It should also be noted that, since enzyme kinetics depend on affinity for substrate, if Fpg is used to detect lesions other than 8-oxoGua, optimal enzyme concentration/incubation time may differ, and should be separately determined. The results of these studies suggest that variation within a laboratory is reduced if care is taken to control these critical parameters. We can recommend conditions, within limits: 0.6-0.8 % agarose (final concentration), 40 min alkaline incubation, and electrophoresis for between 20 and 30 min at around 1 V/cm. Whichever conditions are chosen, they should be precisely maintained, and reported in publications, to facilitate comparison between laboratories.

Within an electrophoresis run, there can be variation, depending on the position of the gel on the platform. This is likely due to local variations in voltage, which are detected by placing a measuring gauge with platinum probes at defined height and spacing on the platform. The variations in voltage—and the variation in % tail DNA of ostensibly identical cell samples—were considerably reduced by introducing mild recirculation of electrophoresis solution using an external pump (Gutzkow et al., 2013).

It is generally recommended that electrophoresis be carried out under refrigeration, so that the temperature of the solution, and the gels, does not rise above 15°C. McKelvey-Martin et al. (1993) showed that, for γ-irradiated and unirradiated lymphocytes, there was little difference in comet appearance with alkaline incubation and electrophoresis at 5°C or 10°C, but a substantial increase in migration occurred at higher temperatures (up to 25°C). This was confirmed by Speit et al. (1999) comparing 4°C and 20°C and was recently also reported by Sirota et al. (2014). Speit et al. (1999) suggest that the higher temperature might be usefully employed to increase the sensitivity of the assay. In any case, a tank with efficient temperature control and monitoring would be a reassuring technical advance.

What level of variation is acceptable? During the recent COMICS project, two partner laboratories carried out similar experiments with TK-6 lymphoblastoid cells treated with 0.25 mM methylmethanesulphonate for 3 h, using exactly the same protocol (Azqueta et al., 2013). We were testing different formats; the standard format of two large gels per slide, or 12 mini-gels per slide, or minigels in an 8 × 12 multi-array format. The CVs were calculated for replicate gels in each of three experiments in each laboratory, and the mean CV was then calculated. Table 1 shows that, for no apparent reason, one laboratory had more variable results than the other. Combining the results from the two laboratories gave CVs of just over 10%—essentially the same for all three formats. Testing the 8 × 12 multiarray format, with X-irradiated cells, Gutzkow et al. (2013) reported a mean CV from three experiments of 26% without recirculation of electrophoresis solution, which reduced to only 7% with circulation. We conclude that a CV of around 10%, for identical cell samples with appreciable damage levels, would be a realistic target (At very low levels of damage, close to 0% tail DNA, the CV will of course be much higher and is no longer meaningful. Conversely, at high levels of damage, as saturation of the assay is approached, the CV will tend to be very small, but again this has little meaning).

**Table 1:** **Coefficients of variation**.

**Format**	**Laboratory A (%)**	**Laboratory B (%)**	**Combined (%)**
2 Gels/slide	4.8	15	12
12 Minigels/slide	7.5	16	12
24 Minigels/GelBond	8.2	13	11

TK-6 cells were treated with 0.25 mM MMS for 3 h. The comet assay was performed using different formats, in two laboratories. The CV was calculated for each of 3 independent experiments in each laboratory; the mean CV is shown here. Also shown is the mean CV for all experiments in both laboratories combined. Data from Azqueta et al. (2013).

## Reference standards

It is sound practice to include reference standard cells in experiments. They should be from a single batch of, for example, human PNMN cells or cultured cells, either untreated (negative controls), or treated with DNA-damaging agent relevant for the particular experiment. Aliquots are then stored under conditions preserving DNA integrity. Slow freezing of PBMN or cultured cells in medium containing serum and 10% dimethylsulphoxide (DMSO) prevents physical damage to DNA; aliquots should be thawed quickly, diluted with PBS or medium and centrifuged without delay to remove the cells from DMSO.

The reference standards serve to monitor performance of the assay. If a particular experiment gives seriously anomalous results for the standards, the results for the samples should be scrutinized, and if necessary the experiment should be repeated. However, minor variations are inevitable, and it is possible to use the reference standard % tail DNA results to improve the precision of sample results.

**Figure 2:**
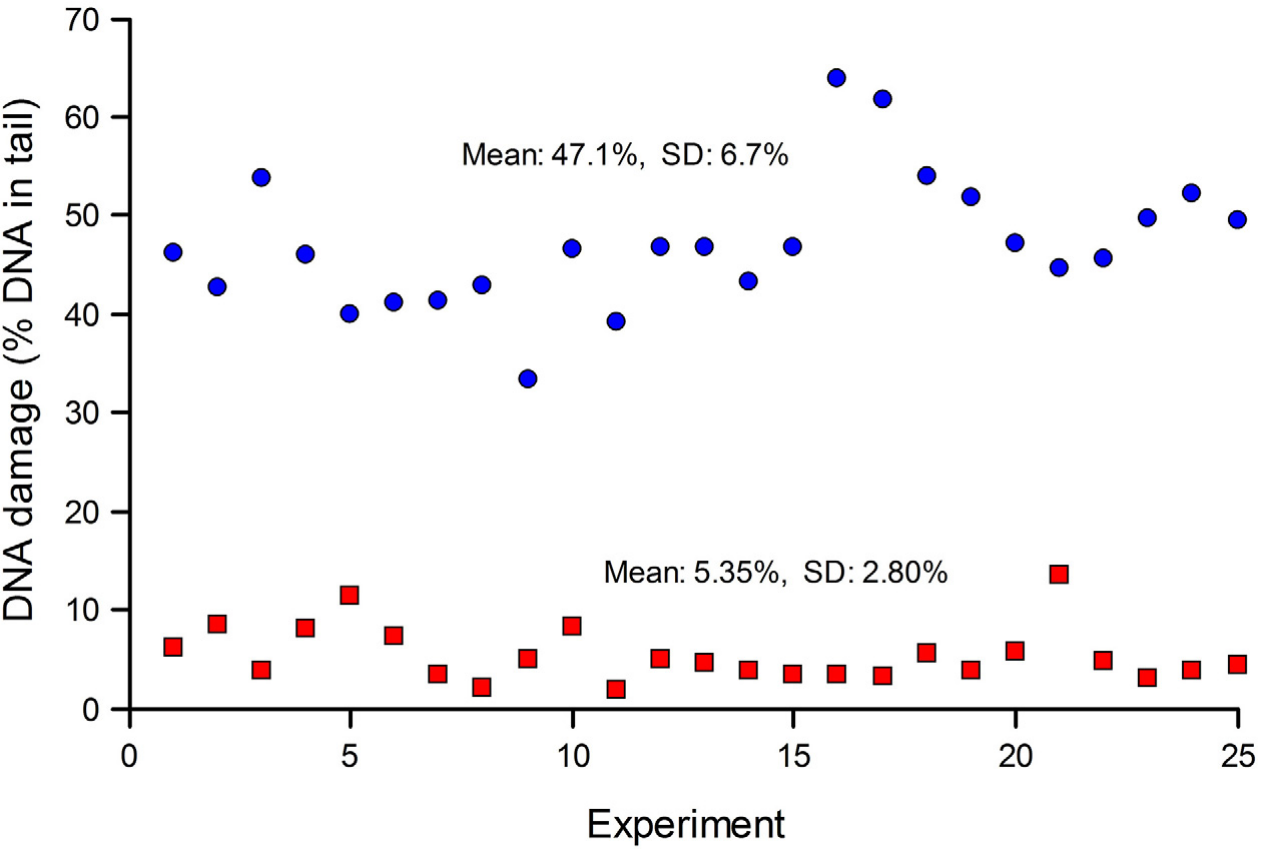
**DNA damage in reference standards, assayed in a series of experiments to measure DNA damage in PBMN cells from a human biomonitoring study**. Aliquots of human lymphocytes from a single batch, either untreated (red squares), or treated with Ro 19-8022 to induce 8-oxoGua (blue circles), were included as standards in each of the experiments alongside the test samples (the results of which are not shown) and analyzed for strand breaks and Fpg-sensitive sites, respectively.

Standards are particularly important when many samples (for example, from a human biomonitoring trial) are analyzed in a series of experiments over an extended period. Figure 2 shows typical results from such a trial; standards—either untreated, or treated with Ro 19-8022 plus light to induce 8-oxoGua—were included in each experiment. The CV for the untreated cells was 52%, while for the treated cells it was 14%. Variation in experiments carried out over a long time period can be expected to be greater than variation within an experiment. Figure 2 also illustrates the point made above, that where levels of damage are close to the limit of detection (0% tail DNA), the relative variation will be greater.

Ideally, reference standards would be internal standards, i.e., cells embedded in the same gel as the sample cells. The problem of distinguishing standard cells from sample cells after electrophoresis has been solved in more than one way, although to the best of our knowledge true internal standards are not employed routinely in any comet assay laboratories. One solution is to pre-label standard cells by incubating them over a cell cycle with bromodeoxyuridine, which is incorporated into DNA in place of thymidine. It can subsequently be recognized by means of a fluorescent-tagged anti-bromouracil antibody (Zainol et al., 2009). When scoring, appropriate (different) filters are used to identify sample and standard cells, and this makes the process of scoring more laborious. A second approach uses as standards cells with a markedly different genome size compared with human—for example, erythrocytes of certain fish species (Brunborg et al., 2014). After scoring all the comets in the gel, they are sorted into two sets according to total comet fluorescence, which is proportional to genome size.

## Normalization

We suggest a procedure for correcting sample data for experimental variation as revealed by reference standards in a series of experiments. As an example, we assume that sample cells have been analyzed for 8-oxoGua (Fpg-sensitive sites) and that data are available from reference standards treated with Ro 19-8022 plus light to induce 8-oxoGua (Positive reference standards should be used; comets from untreated cells are too much affected by high relative variation to be useful for normalization).

- Calculate the median value, M, of net Fpg-sensitive sites (% tail DNA) for the reference cells in all experiments in the series. (Taking the median excludes the anomalously high or low values.)
- With the value of net Fpg-sites (% tail DNA) for the reference cells in a particular experiment X defined as Q, then the correction factor is M/Q.
- Multiply the values of Fpg-sites (mean or median % tail DNA) for samples in experiment X by M/Q.

**Figure 3:**
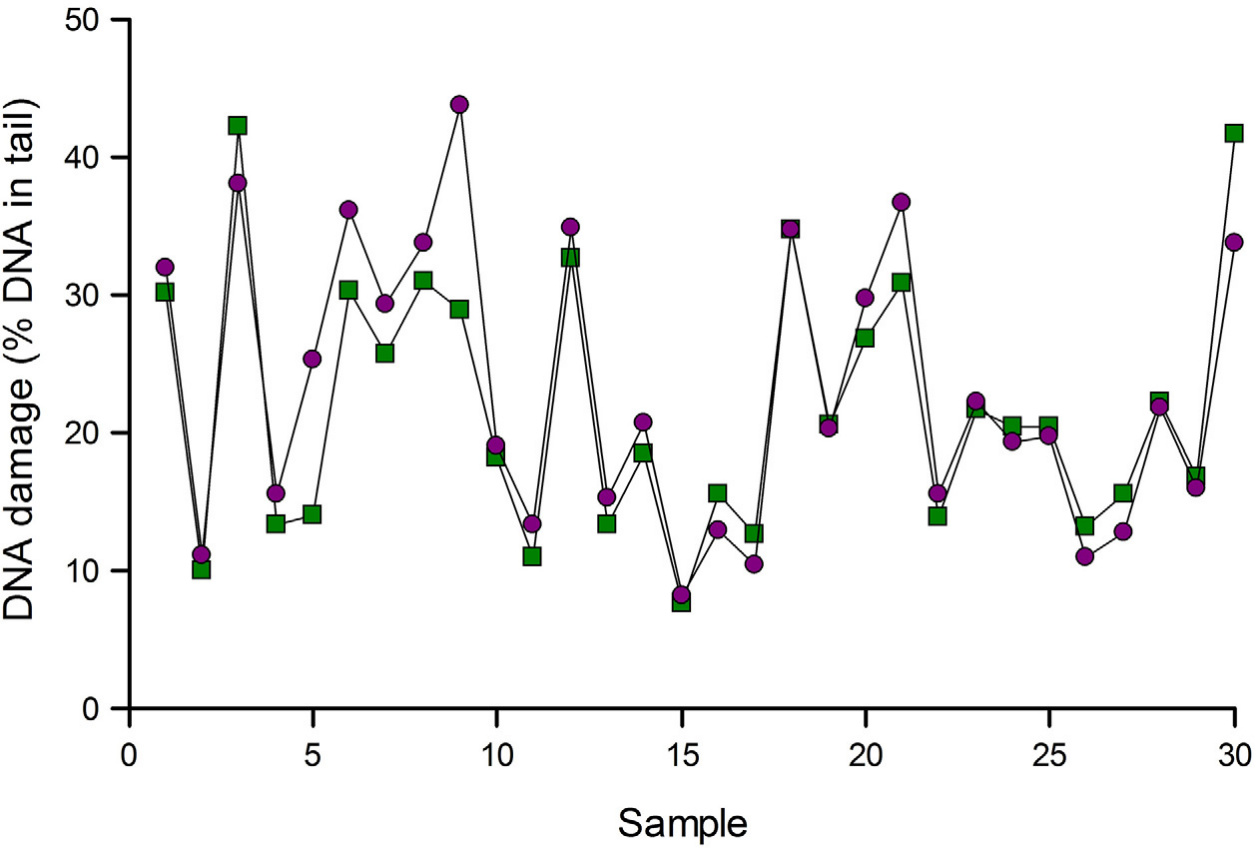
**Normalization of comet assay data**. Results of analysis of 30 lymphocyte samples using Fpg to detect 8-oxoGua were corrected for variation as indicated by reference standards (see text). Data are shown before (green squares) and after (purple circles) normalization. Results for samples 5 and 9 changed substantially after normalization.

Figure 3 gives an example of normalization. Samples of lymphocytes from an intervention study were analyzed for Fpg-sensitive sites. The data were then corrected for variation as indicated by positive reference standards run in the same experiments. In most cases, normalization made little difference, but substantial changes were seen in a few samples, namely 5 and 9 in this set of samples.

## True variation

**Figure 4:**
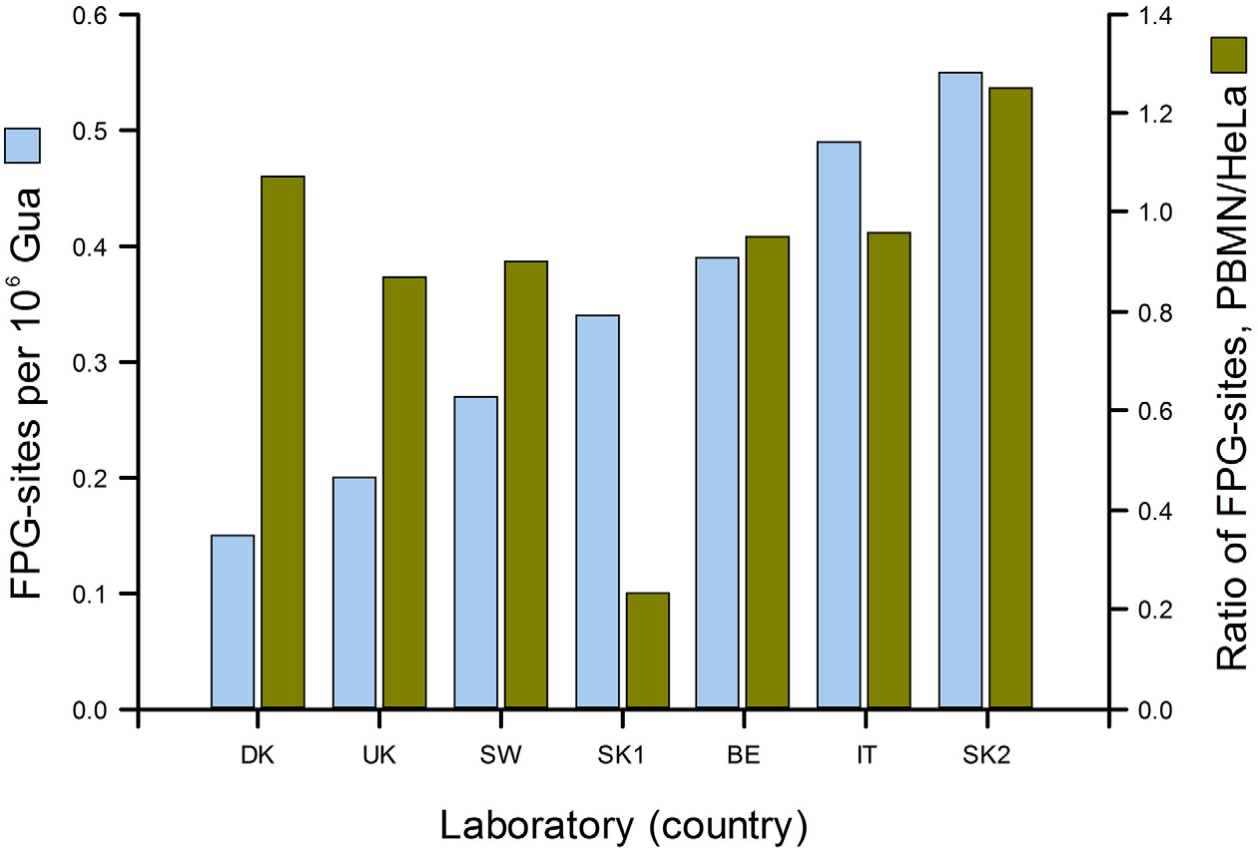
**DNA damage levels in PBMN cells from representative groups of between 8 and 20 healthy subjects in Denmark, United Kingdom, Sweden, Slovakia (two laboratories), Belgium and Italy**. Mean % tail DNA for each laboratory was converted to Fpg-sensitive sites per 10^6^ Gua (light blue bars) using an X-ray calibration curve. To correct for variation between laboratories, mean % tail DNA for PBMN cells was divided by mean % tail DNA for standard HeLa cells (treated in the coordinating laboratory with Ro 19-8022 plus light, and distributed frozen to the partners). The corrected values are shown in dark green (From Collins, 2014 with permission from Elsevier).

Figure 3 gives a good idea of the range of DNA damage levels (in this case, oxidized purines) to be found in an apparently healthy population. In the ESCODD project (ESCODD et al., 2005), we attempted to answer the question whether there are significant differences in DNA damage levels between countries (Figure 4). Partners in this project were asked to collect PBMN cells from healthy volunteers and to measure Fpg-sensitive sites. They also measured Fpg-sites in standard samples of HeLa cells containing 8-oxoGua induced by Ro 19-8022 plus light. The CV of the mean values from the seven laboratories was 43%. When the means were corrected for inter-laboratory variation, by dividing PBMN cell means by the value found for HeLa cells in each laboratory, there was much less variation among the countries—with one exception, which gave a very low value. (This happened to be one of the two laboratories from one country; the other laboratory had a result closer to those of the other countries, and so we assumed that a technical problem in the first laboratory accounted for the low value.) Omitting this outlier, the CV for mean damage levels was only 14%. We can conclude that, in this sample of six countries from different corners of Europe levels of oxidative damage to DNA were quite uniform.

## Conclusions

It is now clearer than ever what are the experimental conditions that most critically influence the % tail DNA recorded for a given cell sample: agarose concentration, electrophoresis time and voltage gradient. For each of these, the effect of variation over a fairly wide range of values is more or less linear. Other factors—lysis time, alkaline incubation time, enzyme concentration and incubation time, electrophoresis temperature—are also important, but optimal conditions can be established which allow a certain amount of latitude; thus, for example, if all enzyme-sensitive sites are detected in 30 min, extending incubation to 45 or 60 min should have no effect. While it is unreasonable to expect all laboratories to adopt exactly the same conditions, they should (a) ensure that whatever conditions are chosen are precisely maintained from experiment to experiment, and (b) specifically describe those conditions in any publication (even though for the overall procedure reference may be made to a previous publication).

The “elephant in the room” is the issue of staining, scoring and image analysis. A conclusion from the first ECVAG trial (Forchhammer et al., 2010) was that most of the observed inter-laboratory variation results from different procedures in staining and analyzing comet images. The concentration of stain can influence comet assay results, as was shown by Olive et al. (1990) in the case of propidium iodide. A wide range of different stains are in use, and little effort has been made to check whether they give comparable results. Comparing different staining procedures, the intercalating dye propidium iodide, minor groove-binding Hoechst 33342 and DAPI showed similar sensitivities (indicated by the slopes of dose-response curves), as did bromodeoxyuridine incorporated into replicating DNA and detected with FITC-conjugated anti-BrdUrd (Olive et al., 1992). The traditional UV light source (mercury vapor lamp) varies in output over time; modern LED light sources are more stable. Various scoring systems are in use. Visual scoring simply categorizes comets into classes [typically from “no tail” (class 0) to “almost all DNA in tail” (class 4)] and computes the overall score for 100 comets, between 0 and 400 arbitrary units. Image analysis, based on a variety of commercial or free software systems, computes mean % tail DNA, tail moment, tail length and other more abstruse properties; most commonly used are % tail DNA and tail moment. (The issue of which parameter to use is addressed in a separate article, by Møller et al., 2014.) Image analysis systems can be manual (i.e., comets being selected by the operator for analysis) or automated. Visual scoring, manual and automated image analysis were compared (Azqueta et al., 2011b), they gave qualitatively similar results in dose response experiments with MMS and H_2_O_2_, but visual scoring overestimated low levels of damage while automated analysis missed highly damaged comets—a defect since rectified.

Sadly, numerous inter-laboratory trials by ESCODD and ECVAG have failed to eliminate variability from the comet assay. There is a need for further ring studies, with even more strictly controlled experimental conditions, distribution of cell samples with different levels of damage, analysis of PBMN cells prepared locally and subjected to defined doses of ionizing radiation, and exchange of the resulting slides between laboratories for re-scoring with different systems—something that has not been done systematically before.

Within a laboratory, experimental variation of around 10% is acceptable, though this will depend on the damaging agent: cells treated with ionizing radiation are likely to show damage responses that are more homogeneous than when treated with chemicals, since cellular metabolic responses are not involved, and so the variation between gels or samples should be relatively low.

### Conflict of interest statement

The authors declare that the research was conducted in the absence of any commercial or financial relationships that could be construed as a potential conflict of interest.
